# E2F1/TS Immunophenotype and Survival of Patients with Colorectal Cancer Treated with 5FU-Based Adjuvant Therapy

**DOI:** 10.1007/s12253-016-0043-z

**Published:** 2016-01-29

**Authors:** Violetta Sulzyc-Bielicka, Pawel Domagala, Dariusz Bielicki, Krzysztof Safranow, Wojciech Rogowski, Wenancjusz Domagala

**Affiliations:** Department of Clinical Oncology, Pomeranian Medical University, Szczecin, Poland; Department of Pathology, Pomeranian Medical University, Unii Lubelskiej 1, 71-252 Szczecin, Poland; Department of Gastroenterology, Pomeranian Medical University, Szczecin, Poland; Department of Biochemistry and Medical Chemistry, Pomeranian Medical University, Szczecin, Poland; Department of Oncology, University of Warmia and Mazury, Olsztyn, Poland

**Keywords:** Colorectal cancer, E2F1, Thymidylate synthase, 5FU-based therapy

## Abstract

The predictive value of thymidylate synthase (TS) expression alone for 5FU-based treatment of colorectal cancer (CRC) has not been clinically confirmed. Little is known on the association of expression of E2F1, which controls the transcription of genes encoding proteins engaged in DNA synthesis including *TS*, and survival of patients with CRC. The purpose of this study is to assess the correlation between expression of both E2F1 and TS in CRCs and survival of patients administered adjuvant 5FU-based chemotherapy, in order to find a better predictor of treatment outcome than expression of TS or E2F1 alone. Nuclear TS and E2F1 were detected by immunohistochemistry in tissue microarrays from 190 CRCs (Astler-Coller stage B2 or C). Multivariate analysis identified significant association of the combined E2F1+TS+ immunophenotype with worse OS (HR = 3,78, *P* = 0,009) and DFS (HR = 2,30, *P* = 0,03) of patients with colon cancer. There were significant differences between E2F1+TS+ and E2F1-TS- Kaplan-Meier survival curves in relation to DFS (*P* = 0.008) and OS (*P* = 0.01). About 37 and 31 % difference in 3-year DFS and OS respectively were seen between patients with E2F1+TS+ vs. E2F1-TS- colon cancer immunophenotype. The E2F1+TS+ immunophenotype may be a marker of poor prognosis (the worst DFS and OS) of patients with colon cancer treated with 5FU-based adjuvant therapy. A subgroup of patients with this immunophenotype may require different and perhaps more aggressive treatment than 5FU-based chemotherapy. Thus, the combined E2F1/TS immunophenotype could be a potential indicator of colon cancer sensitivity to 5FU.

## Introduction

Adjuvant 5-fluorouracil (5FU)-based chemotherapy in stage III colorectal cancer (CRC) decreases the frequency of cancer relapse and reduces the risk of cancer associated deaths by 30 % [1, 2]. In stage II colorectal cancer, the benefit of adjuvant chemotherapy is smaller, improving the 5-year survival rate by 3–6 % [3]. In advanced CRC approximately 23 % of patients will respond to 5FU treatment combined with leucovorin [4]. It follows that a sizable percentage of CRC patients will not benefit from adjuvant 5FU-based therapy but will experience toxic side effects of the therapy and unnecessary costs [5]. Therefore, it is essential to know precisely which patients will respond to this kind of therapy.

5-fluorouracil has been used in cancer treatment for the past 40 years. 5FU is an uracil analog and a pro-drug [6], which undergoes a biotransformation to pharmacologically-active metabolites. The main target of 5FU is thymidylate synthase (TS) because 5-fluorodeoxyuridine-5′-monophosphate (FdUMP), a 5FU metabolite, binds TS and forms a stable ternary complex that prevents DNA synthesis [6]. Studies using CRC cell lines have suggested the predictive importance of TS expression with regard to 5FU-based chemotherapy [7]. However, clinical trials of predictive/prognostic significance of TS in CRC patients have reported discrepant findings [8–10]. Therefore it was concluded that TS expression alone could not be used in clinical practice as a predictive marker [11]. Recent findings clearly indicate that markers associated with the cell cycle can help to identify subgroups of CRCs sensitive to 5FU treatment [12–14]. The E2F1 transcription factor could be one of such markers because: (1) it regulates the expression of TS and other enzymes necessary for DNA synthesis, (2) deregulation of *E2F1* is one of the initial events in colorectal carcinogenesis [15] and analysis of protein products of genes associated with carcinogenesis can help to clarify their biological role in CRC, (3) high TS expression in tumor cells may be induced by E2F1 overexpression [16, 17], (4) E2F1 may bind to the *TS* gene promoter region [17], (5) high correlation between *TS* gene and *E2F1* gene expression was found [18], (6) most CRCs show varying degrees of E2F1 expression [19].

E2F1 belongs to a family of eight (E2F1-8) transcription factors which regulate the cell cycle [20]. The activation of E2F transcription factors through their release from phosphorylated pRB complexes leads to transcription of over 1200 genes [21] including *TS* and other genes involved in DNA synthesis [17, 22, 23]. E2F1 overexpression may promote proliferation or enhance apoptosis depending on the level of E2F1 deregulation and the cell context background [20].

Very few studies have addressed the issue of E2F1 expression and survival of patients with CRC treated with chemotherapy. These reports assessed small groups of patients at various stages of advanced colorectal cancer with discrepant results [19, 24]. Hence, on one hand, the predictive value of assessment of TS expression alone for 5FU-based treatment of CRC has not been clinically confirmed. On the other hand, although E2F1 seems to be one of several potential predictors for response to 5FU-based therapy, because it controls the transcription of genes encoding proteins engaged in DNA synthesis including *TS*, little is known on the association of E2F1 protein expression and survival of patients with CRC.

Therefore, to find a better predictor of treatment outcome than TS or E2F1 alone, the aim of this study was to assess the correlation between immunohistochemically determined nuclear expression of both E2F1 and TS in CRCs and overall survival (OS) and disease free survival (DFS) of patients administered adjuvant 5FU-based chemotherapy.

## Materials and Methods

### Patients

The study group consisted of 190 consecutive patients (mean age 59.3 ± 10.5) who met the following criteria: (1) had undergone potentially curative colorectal resection for sporadic CRC (defined as an absence of relevant family history at the time of admission to the hospital). The surgical operations consisted of either a resection with lymphadenectomy or a total mesorectal excision for rectal carcinomas; (2) distant metastases were excluded on preoperative liver ultrasonography, chest X-ray, and during intraoperative exploration; (3) histopathologic diagnosis of invasive adenocarcinoma Astler-Coller stage B2 or C without involvement of resection margins was established; (4) had no chemotherapy prior to the operation; (5) received identical adjuvant 5FU-based therapy. All patients received the same adjuvant chemotherapy regimen (6 cycles of 5 day courses of 425 mg/m^2^ 5FU administered intravenously plus 20 mg/m^2^ leucovorin). The cycles were repeated every 4 weeks). A total of 40 patients with rectal tumors received postoperative radiotherapy (50,4 Gy). Of the 77 Astler-Coller stage B2 tumors, 36 were rectal cancers and, of these, 14 (38.9 %) patients received preoperative and 10 (27.8 %) received postoperative radiotherapy. Tissues from the former were obtained from post-therapeutic resection specimens. Since there were no statistically significant differences in TS and E2F1 expression between patients with rectal cancer who did or did not undergo preoperative radiotherapy [TS (51.6 % vs. 61.3 % respectively, *P* = 0.38), E2F1 (22.6 % vs. 35.5 % respectively, *P* = 0.24)] the former were included in the study. Table 1 lists the clinico-pathological characteristics of the 190 tumors and patients. The Research Ethics Committee of the Pomeranian Medical University approved this study (KB-0080/101/09).

**Table 1:** Characteristics of the study group (*n* = 190)

Parameter	*n*
Age (years)	
≤60	100
>60	90
Sex	
Females	86
Males	104
Grade	
G1+G2	100
G3^a^	90
Astler-Coller stage	
B2	77
C	113
Site	
Rectum	95
Colon	95
Radiotherapy (rectal tumors) (*n* = 73)	
Preoperative	31
Postoperative	40
Unknown	2

^a^Including mucinous carcinoma

The time from surgery until the time of death due to cancer or to last known follow-up (≥36 months from the operation) was regarded as OS, and the time until the first appearance of metastasis or local recurrence was regarded as DFS. The median follow-up was 51 months (mean 51.0 ± 23.8; range 7–120). During the follow-up, 44 of the 190 (23.2 %) patients died of cancer. Recurrences were found in 73 patients. Four patients died for reasons unrelated to cancer and were treated as censored observations.

### Immunohistochemistry

Tumor tissue was fixed in buffered 10 % formalin and embedded in paraffin. Sections (4 μm thick) were stained with hematoxylin and eosin for histopathological diagnosis. Tissue microarrays (TMs) were constructed as previously described [13, 14]. Slides with TMs were deparaffinized and rehydrated, and endogenous peroxidase activity was blocked. Slides were immersed in pH 9.0 buffer and heat induced antigen retrieval was performed in a pressure cooker (Pascal, DakoCytomation). Monoclonal TS-106 (Chemicon, Temecula, CA) antibody (dilution 1:50, incubation time 30 min) and monoclonal E2F1 antibody (Santa Cruz Biotechnology, USA, dilution 1:100, incubation time 30 min) were used, and the TMs were immunostained using the Dako Envision kit according to the manufacturer’s instructions (Envision ™ + peroxidase anti-mouse polymer labeled with horseradish peroxidase – Dako Co., Carpinteria, CA). The reaction was developed with a diaminobenzidine substrate – chromogen solution and slides were counterstained with hematoxylin. Appropriate positive and negative controls were run. The immunohistochemical procedure for all 190 tumors was run at the same time under identical conditions because tumor tissue cores were contained in only four slides. Immunohistochemistry for assessing the presence or absence of DNA mismatch repair (MMR) proteins was performed on 180 CRCs (10 tumors could not be assessed due to insufficient amount of tissue) as described in [14]. Immunohistochemistry with antibodies directed against MMR proteins has been regarded as an equivalent for microsatellite instability (MSI) testing [25]. Loss of MMR protein expression was defined as complete absence of nuclear staining in the presence of positive staining of stromal cells.

### Scoring

Tumor cores were independently assessed by 2 observers (PD and WD) who were blinded to clinical and pathological data. In cases of disagreement, the result was reached by consensus. Semi-quantitative evaluation of immunostained sections was done using well defined and tested histo-score system [26]. Of three most frequently applied scoring systems used in the literature to determine the expression status of immunohistochemically assessed proteins. (i.e., intensity score, pattern score or both combined), we used intensity & pattern score because it seems to be most reliable and proved to be useful and reproducible in assessment of immunohistochemical staining [26–28]. Both intensity (0–3) and pattern (1–6) scores were assessed. Each intensity score was multiplied by its corresponding pattern score (1 = 0–4 % of positive tumor cells; 2 = 5–19; 3 = 20–39; 4 = 40–59; 5 = 60–79; 6 = 80–100 %) and these grades were added to give the final histo–score (a minimum value of zero and a maximum value of 18). In order to reach the histo-score all tumor cells in a core of the TM were counted. A histoscore value of 4 was adopted as a cut-off for stratification of E2F1expression into low (≤4, E2F1-) and high (>4, E2F1+), because the histogram of E2F1 values showed a local minimum at this point that clearly divided the study population into two subgroups. Similarly as in [14], stratification of TS expression into low (<2, TS-) and high (≥2, TS+) was based on a local minimum in the histoscore histogram.

### Statistics

Associations between the presence of high TS or E2F1 expression in tumors and other categorical variables were analyzed with the Fisher exact test. The Kaplan–Meier method was used for the univariate survival analysis, and the differences between groups were assessed by the log-rank test. A Cox proportional hazards model was used for univariate and multivariate analyses of factors associated with OS and DFS. The independent variables included in the model were: age, gender, tumor site (rectum vs. colon), Astler–Coller stage (C vs. B2), histological grade (G3+mucinous vs. G1+G2), loss of MMR proteins, presence of high TS expression and presence of high E2F1 expression. A *p* < 0.05 was considered statistically significant. STATISTICA version 10 (StatSoft Inc., Tulsa, USA) was used for the statistical analysis.

## Results

### E2F1 and TS Expression and Clinico-Pathologic Parameters

**Fig. 1:**
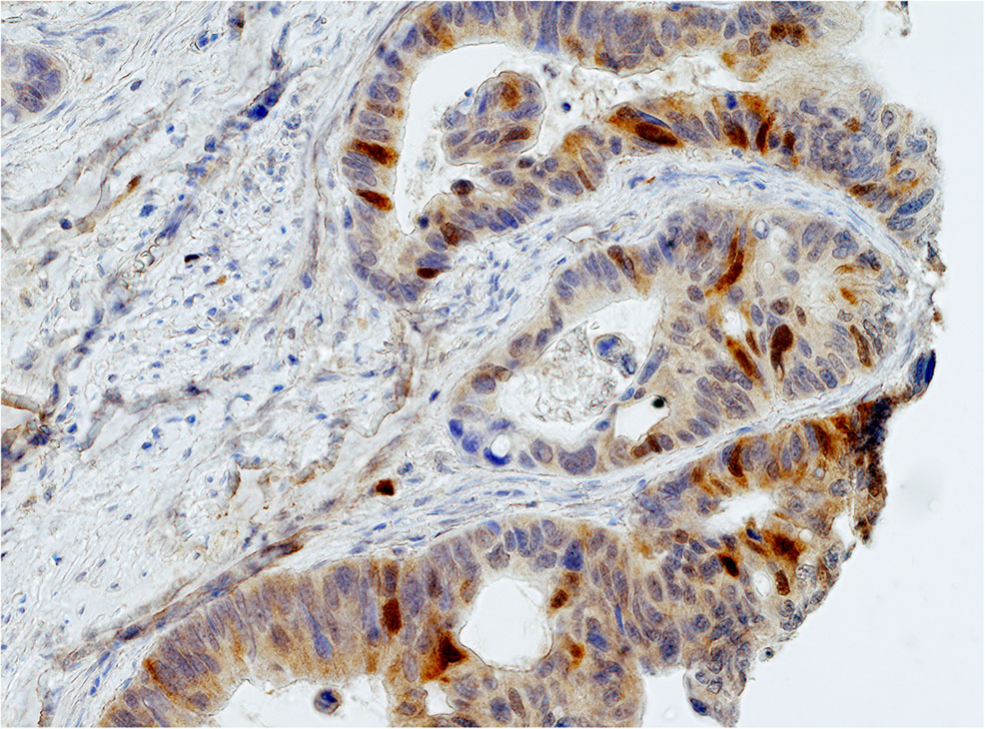
Nuclear expression of TS (*brown*) in a colon carcinoma

**Fig. 2:**
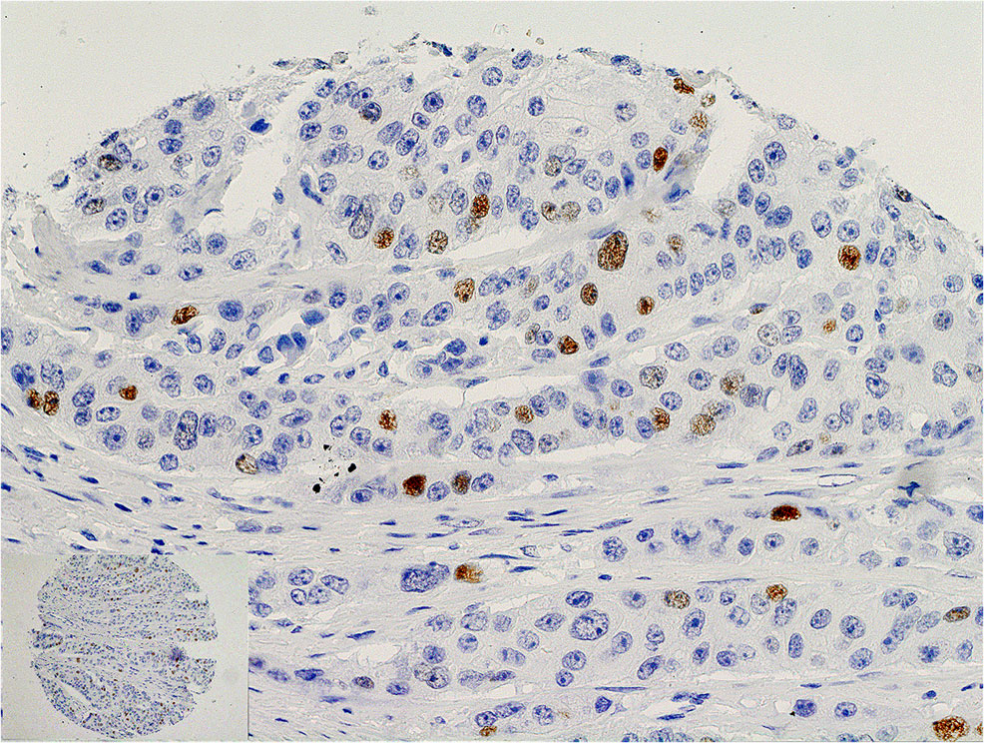
Nuclear expression of E2F1 (*brown*) in a colon carcinoma. This is higher magnification of the upper portion of a core shown in an inset (*lower left corner*)

TS expression (Fig. 1) and E2F1 expression (Fig. 2) were seen in nuclei of cancer cells. Mean and median histoscore values were 3.7 and 4.0, respectively for E2F1, and 4.0 and 3.0, respectively for TS. High TS (TS+) expression (histoscore TS ≥ 2) in tumor cell nuclei was noted in 128 out of 190 patients (67.4 %). High E2F1 (E2F1+) expression (histoscore E2F1>4) was seen in 36.8 % of patients (70 out of 190 patients). The E2F1+TS+ immunophenotype was found in 51 (26.8 %) patients, E2F1+TS- in 19 (10.0 %), E2F1-TS+ in 77 (40.5 %) and E2F1-TS- in 43 (22.6 %) patients. Loss of MMR proteins was found in 31 out of 180 (17.2 %) CRCs.

We investigated associations of E2F1 and TS expression with the following parameters: age and gender of patients, tumor grade, stage, and tumor site. High TS expression in tumor cell nuclei was seen more often in tumors located in the colon compared with those in the rectum (76.8 % vs. 57.9 %, *P* = 0.008). High E2F1 expression was more frequent in tumors with B2 stage compared with C stage (46.8 % vs. 30.1 %, *P* = 0.02). It was also more frequent in females than in males although the difference did not reach statistical significance (44.2 % vs. 30.8 %, *P* = 0.07). There was no significant association between high E2F1 and high TS expression (high E2F1 expression was present in 30.7 % of patients with low TS and in 39.8 % of those with high TS, *P* = 0.26). There was no significant correlation between MSI status and E2F1/TS positivity (*p* = 0.66).

### Survival of Patients with CRC Defined by E2F1 and TS Expression

Multivariate analysis involving sex, age, Astler-Coller stage, tumor grade, tumor site, loss of MMR proteins and separately E2F1 and TS expression in all patients (*n* = 180) identified significant associations only for Astler-Coller stage (C vs. B2) which was significantly associated with worse DFS (HR = 3.01, 95 % CI = 1.66–5.46, *P* = 0.0003) and OS (HR = 3.24, 95 % CI = 1.49–7.03, *P* = 0.003), male sex associated with worse OS (HR = 2.05, 95 % CI = 1.04–7.03, *P* = 0.04) and loss of MMR which was significantly associated with better DFS (HR = 3.71, 95 % CI = 1.74–7.91, *P* = 0.0007). The association of the loss of MMR proteins with OS did not reach statistical significance. However, multivariate analysis (Table 2) involving the above mentioned parameters and the combined E2F1+TS+ immunophenotype (high E2F1 and high TS expression) identified in addition to Astler-Coller stage (*P* = 0.0001 for DFS and *P* = 0.002 for OS), sex (*p* = 0.04 for OS), grade (*p* = 0.048 for OS) and loss of MMR (*p* = 0.02 for DFS), significant association of E2F1+TS+ immunophenotype with worse OS (HR = 2.35, 95 % CI = 1.21–4.58, *P* = 0.01) and DFS (HR = 1.73, 95 % CI = 1.01–2.98, *P* = 0.047).

**Table 2:** Colorectal cancer. Multivariate analysis of relations between expression of E2F1+TS+^1^, and other clinico-pathological parameters, and OS and DFS (*n* = 180)

Parameters	DFS		OS	
	Hazard ratio (95 % CI)	*P*	Hazard ratio (95 % CI)	*P*
Male Sex	1.60	0.07	2.03	0.04
	0.95–2.68		1.03–3.99	
Age	1.01	0.67	1.01	0.97
	0.98–1.03		0.97–1.03	
Astler-Coller C	3.13	0.0001	3.35	0.002
	1.73–5.66		1.55–7.22	
Grade G3	1.55	0.09	1.93	0.048
	0.94–2.53		1.01–3.68	
Site rectum	1.05	0.84	1.03	0.93
	0.64–1.74		0.54–1.94	
E2F1+TS+	1.73	0.047	2.35	0.01
	1.01–2.98		1.21–4.58	
MSI	0.34	0.02	0.48	0.17
	0.13–0.85		0.17–1.38	

^1^ *E2F1*+ high expression of E2F1, *TS*+ high expression of TS

Because rectal cancers received pre- or postoperative radiotherapy and colon cancers did not, in order to see whether the results might depend on the site of the tumor, patients with tumors localized to the colon and rectum were analyzed separately.

### Colon

Multivariate analysis involving sex, age, Astler-Coller stage, tumor grade, loss of MMR proteins and separately E2F1 and TS expression in patients with colon cancer (*n* = 90) identified only significant correlation of TS+ (HR = 3.96, 95 % CI = 1.19–13.22, *P* = 0.03), Astler Coller C stage (HR = 3.01, 95 % CI = 1.29–7.02, *P* = 0.01) and loss of MMR proteins (HR = 0.20, 95 % CI = 0.04–0.86, *P* = 0.03) with DFS. However, multivariate analysis involving the above mentioned parameters and the combined E2F1+TS+ immunophenotype identified significant association of E2F1+TS+ immunophenotype with worse OS (HR = 3.78, 95 % CI = 1.38–10.33, *P* = 0.009) and with worse DFS (HR = 2.30, 95 % CI = 1.08–4.90, *P* = 0.03) (Table 3). This association remained significant (OS *P* = 0.006, DFS *P* = 0.02) when only MSI negative cases were analyzed.

**Table 3:** Colon cancer. Multivariate analysis of relations between expression of E2F1+TS+^1^, and other clinico-pathological parameters, and OS and DFS (*n* = 90)

Parameters	DFS		OS	
	Hazard ratio (95 % CI)	*P*	Hazard ratio (95 % CI)	*P*
Male sex	1.54	0.27	2.29	0.11
	0.72–3.30		0.82–6.41	
Age	1.10	0.63	1.00	0.88
	0.97–1.04		0.96–1.05	
Astler-Coller C	2.77	0.02	2.59	0.08
	1.19–6.45		0.90–7.42	
Grade G3	1.65	0.18	1.18	0.74
	0.79–3.44		0.45–3.09	
E2F1+TS+	2.30	0.03	3.78	0.009
	1.08–4.90		1.38–10.33	
MSI	0.23	0.047	0.19	0.11
	0.05–0.98		0.02–1.44	

^1^ *E2F1*+ high expression of E2F1, *TS*+ high expression of TS

**Fig. 3:**
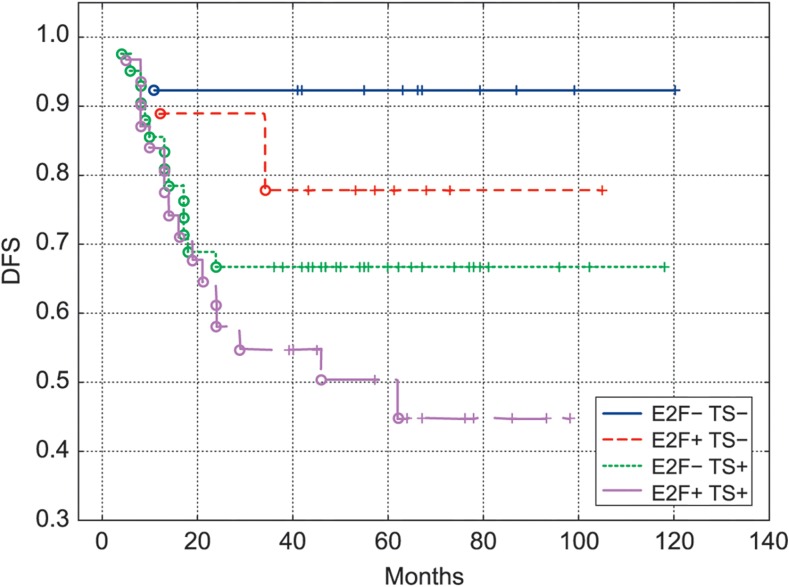
DFS of patients with colon cancer (*n* = 95) treated with adjuvant chemotherapy, categorized according to the E2F1/TS expression. E2F1+TS+ curve differs significantly from E2F1-TS- curve (*P* = 0.008) but not from the other curves (*P* = 0.12; *P* = 0.18; for E2F1+TS-, E2F1-TS+ curves respectively)

**Fig. 4:**
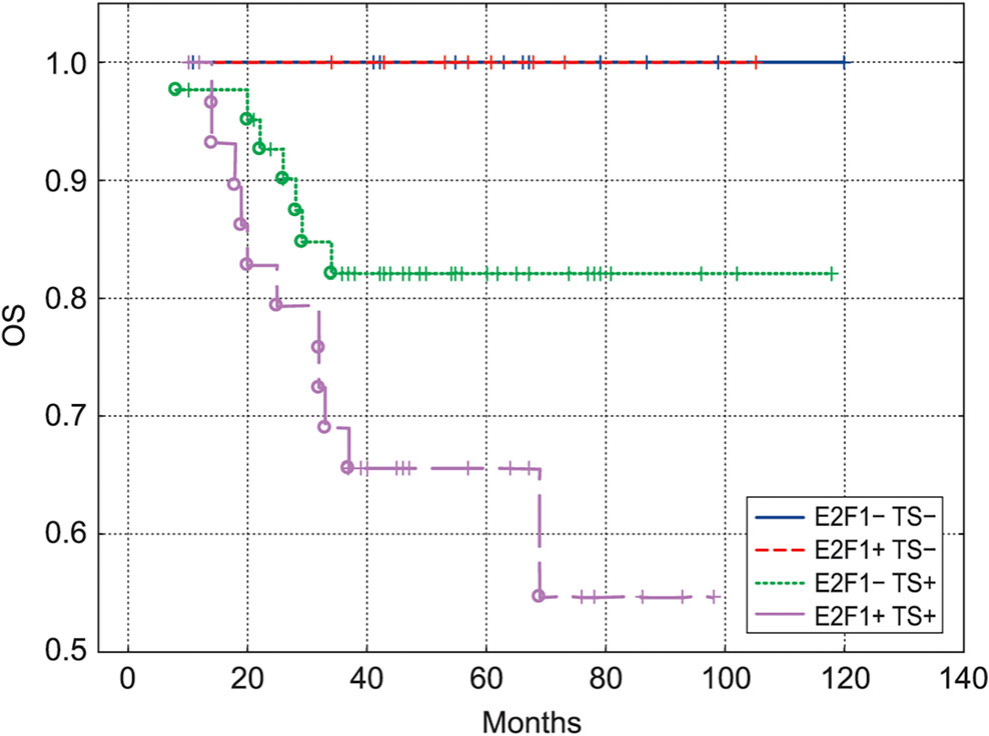
OS of patients with colon cancer (*n* = 95) treated with adjuvant chemotherapy, categorized according to the E2F1/TS expression. E2F1+TS+ curve differs significantly from E2F1-TS- curve (*P* = 0.01) and from E2F1+TS- curve (*P* = 0.049) but not from E2F1-TS+ curve (*P* = 0.07)

Kaplan-Meier survival curves confirmed the worst DFS (Fig. 3) and OS (Fig. 4) of patients with colon cancer exhibiting E2F1+TS+ immunophenotype. There were significant differences between E2F1+TS+ and E2F1-TS- curves in relation to DFS (*P* = 0.008) and OS (*P* = 0.01). For the combined E2F1/TS status, the 3-year DFS rates were 92 % in 13 patients with both low E2F1 and low TS tumors, 55 % in 31 patients with both high E2F1 and high TS tumors, 78 % in 9 patients with high E2F1 and low TS tumors and 67 % in 42 patients with low E2F1 and highTS tumors (Fig. 3). For the combined E2F1/TS status, the 3-year OS rates were 100 % in 13 patients with both low E2F1 and low TS tumors, 69 % in 31 patients with both high E2F1 and high TS tumors, 100 % in 9 patients with high E2F1 and low TS tumors and 82 % in 42 patients with low E2F1 and highTS tumors (Fig. 4). About 37 and 31 % diference in 3-year DFS and OS respectively were seen between patients with E2F1+TS+ colon cancer immunophenotype as compared to E2F1-TS- immunophenotype (Figs. 3 and 4).

### Rectum

In patients with rectal cancer, (*n* = 90) there was no association between the E2F1/TS immunophenotype and DFS or OS (*P* = 0.94 and *P* = 0.76 respectively for multivariate analysis).

There was significant interaction between the site of the tumor (colon vs. rectum) and the E2F1+TS+ immunophenotype with regard to an association with OS (*P* = 0.047) but significance of interaction was not achieved with regard to DFS (*P* = 0.15) in multivariate analysis. These findings indicate that only patients with colon cancer (but not those with rectal cancer) treated with adjuvant 5FU could be stratified into better or worse OS prognostic subgroups by TS and E2F1 expression.

## Discussion

Despite numerous studies and clinical trials analysing predictive/prognostic significance of TS in CRC patients, TS expression alone has not been recommended in clinical practice as a predictive marker of 5FU-based treatment [11]. However, recent report clearly indicates that TS in combination with important regulators of the cell cycle (p53, p21^WAF1^) can better identify subgroups of CRCs sensitive to 5FU treatment than TS alone [14]. For reasons explained in the Introduction we hypothesized that the E2F1 transcription factor, as important cell cycle associated protein, together with TS could be a better predictor of treatment outcome than TS or E2F1 alone. It has been shown that E2F1-overexpressing cells had increased *TS* expression and experimental studies indicated that E2F1 induces various genes associated with S phase, including *TS* [16, 17].

In the present study we have compared E2F1/TS immunophenotype of CRCs with survival of patients treated with 5FU-based therapy. The results suggest that high E2F1 expression when combined with high TS expression predicts poor prognosis of patients with colon cancer treated with 5FU-based regimen. E2F1+TS+ immunophenotype was associated with poor DFS and OS whereas E2F1-TS- immunophenotype was associated with excellent survival. It seems that the worst survival of patients with colon cancer treated with 5FU-based regimen and exhibiting high TS expression may be attributed to TS expression induced by high level of E2F1 (E2F1+TS+ immunophenotype). Thus, our results are in line with studies which found that high *E2F1* expression in CRC cell lines was associated with low sensitivity to 5FU [29, 30] and that high TS expression was associated with 5FU resistance [31]. Generally, irrespective of E2F1 expression, tumors exhibiting high TS expression in our study were refractory to treatment with 5FU-based therapy. However, some TS+ tumors were E2F1+ and others were E2F1- indicating different mechanisms used by tumor cells to increase TS level and acquire resistance to treatment.

*E2F1*’s transcriptional regulation and responses of its downstream targets constitute a complex issue. E2F1 levels are dynamically and differentially regulated during the cell cycle because *E2F1* promoter is equipped with sites for both repression and activation [32]. Therefore, E2F1 may act as an oncogene or a tumor suppressor depending on tumor cell context i.e., *E2F1* is mediating either cell proliferation and growth or tumor suppresion and apoptosis [20, 33, 34]. Although E2F1 induces apoptosis and decreased proliferation in cell lines [35, 36] and tumor tissue in CRCs [37], there is evidence that high E2F1 expression may be associated with CRC progression and metastasis [18, 38, 39]. Perhaps, in order to promote apoptosis or proliferation and survival of cells, different threshold levels of E2F1 are required for differential gene transactivation of its target gene promoters [20]. We speculate therefore that somewhat better survival of patients with tumors exhibiting E2F1+TS- immunophenotype, as compared to the worst survival associated with E2F1+TS+ immunophenotype, may reflect dual activity of E2F1 and point to the important role of increased TS expression which when induced by E2F1 may promote proliferation of tumor cells and resistance to 5FU-based treatment. E2F1 overexpressing human fibrosarcoma cells in culture had increased TS levels and were resistant to 5FU [16], which is consistent with colon cancers exhibiting E2F1+TS+ immunophenotype in our study. Interestingly, Banerjee et al. [16] have shown that cells overexpressing E2F1 were more sensitive to etoposide, doxorubicin, and SN-38, the active metabolite of irinotecan, despite being resistant to 5FU. It has to be shown whether colon cancers with E2F1+TS+ immunophenotype, may behave smilarly i.e., may be more sensitive to topo I inhibitors. There are only a few reports on the association between E2F1 expression alone and survival in CRC patients. These studies were based on small heterogeneous groups of patients and yielded conflicting results [19, 24].

TS is one of the basic enzymes participating in DNA synthesis, and serves as the molecular target for 5FU [6]. The results of studies on prognostic/predictive significance of TS expression alone in patients with CRC have been inconsistent and contradictory [11]. It has been suggested that resistance to 5FU chemotherapy cannot be assigned solely to TS or p53 expression [40] rather, markers associated with the cell cycle should be included in the search for predictive markers of benefit for 5FU-based chemotherapy in CRC [12]. As part of its important role in the cell cycle regulation, E2F1 activates the *TS* promoter and therefore it may influence the results of 5FU-based treatment of CRC [17, 22].

There is only one report in the literature in which expression of *TS* mRNA and *E2F1* mRNA were compared in the same CRCs [18]. In that study increased *E2F1* and *TS* mRNA expression was noted in 14 % of 23 CRCs. We found high expression of both E2F1 and TS protein in 27 % of 190 CRCs but we noted intertumoral heterogeneity with respect to the combined E2F1/TS immunophenotype. An association of *E2F1* expression with the level of TS was found in primary [18] and metastatic CRCs [39]. However, in another report based on 17 primary CRCs the level of E2F1 protein expression did not correlate with TS expression [24]. The results of our study suggest that this may be the result of intertumoral heterogeneity of E2F1/TS immunophenotype.

We did not find an association between E2F1/TS immunophenotype and OS or DFS of patients with rectal cancer. Although there were no statistically significant differences in TS and E2F1 expression between patients with rectal cancer who did or did not undergo preoperative radiotherapy, one cannot exclude the influence of preoperative radiotherapy on the expression of E2F1 and *E2F1*-dependent genes including *TS*. This aspect requires further study. We would like to mention also some limitations of this study. Although all patients received the same type of adjuvant chemotherapy, the series of patients in our study is relatively small and heterogenous. However, the associations we found were strong and highly significant.

We conclude that the E2F1+TS+ immunophenotype may be a marker of poor prognosis (the worst DFS and OS) in patients with colon cancer treated with 5FU-based adjuvant therapy. It seems that subgroup of patients with this immunophenotype may require different and perhaps more aggressive treatment than 5FU-based chemotherapy. Thus, the combined E2F1/TS immunophenotype could be a potential indicator of colon cancer sensitivity to 5FU. Our results also suggest that one way to improve the results of treatment of TS+ colon cancer may be to look for drugs targeting E2F1 or downstream genes of E2F1 other than TS. However, our results are based on a retrospective study and require confirmation on larger number of colon cancer patients and in prospective randomized trials.
